# Osteoarthritis of the talonavicular joint with pseudarthrosis of the navicular bone: a case report

**DOI:** 10.1186/1752-1947-5-547

**Published:** 2011-11-08

**Authors:** Noriyuki Kanzaki, Takayuki Nishiyama, Takaaki Fujishiro, Shinya Hayashi, Yoshiyuki Takakura, Yoshinori Takakura, Masahiro Kurosaka

**Affiliations:** 1 Department of Orthopedic Surgery, Kobe University Graduate School of Medicine, 7-5-1, Kusunoki-Cho, Tyuo-Ku, Kobe, 650-0017, Japan; 2 Department of Orthopedic Surgery, Takakura Orthopedic and Sports Clinic, 5-4-21, Tokui-Cho, Nada-Ku, Kobe, 657-0033, Japan

## Abstract

**Introduction:**

Osteoarthritis of the talonavicular joint caused by inflammatory, degenerative, and post-traumatic arthritis has been commonly described, and isolated arthrodesis for talonavicular joint has usually been performed for such conditions. However, arthritis accompanied by pseudarthrosis of the navicular bone is an extremely rare case, and to the best of our knowledge, isolated arthrodesis for this situation has not been previously described in any published reports.

**Case presentation:**

The patient was a 39-year-old Japanese man. He had complained of pain in his left middle foot since a fall from his motorcycle six months previously. Radiographs and computed tomography (CT) scans revealed pseudarthrosis of the navicular bone. MRI indicated mild arthritic change in the talonavicular joint and avascular necrosis of the navicular bone. We performed an isolated arthrodesis of the talonavicular joint with two 6.5 mm cancellous screws. One year after the operation, radiographical bone union had been obtained, and the patient reported no pain and complete satisfaction with the result.

**Conclusions:**

Isolated talonavicular arthrodesis is one of the effective procedures for the treatment of traumatic talonavicular arthritis with pseudarthrosis of the navicular bone both in providing pain relief and functional improvement.

## Introduction

Osteoarthritis of the talonavicular joint caused by inflammatory arthritis such as rheumatoid arthritis and pes valgus deformity has been commonly described [1,2], but osteoarthritis occurring as a result of fracture of the navicular bone is rare [3]. Arthritis accompanied by pseudarthrosis of the navicular bone is an extremely rare case.

Isolated arthrodesis for talonavicular joint has usually been performed for pes valgus deformities, congenital deformities, neuromuscular diseases, and arthritic conditions, including inflammatory, degenerative, or post-traumatic arthritis [3-11].

We report a case of osteoarthritis of the talonavicular joint accompanied by pseudarthrosis of the navicular bone, which was treated with isolated arthrodesis for the talonavicular joint.

## Case presentation

A 39-year-old Japanese man sustained an injury to his left foot. He had fallen from his motorcycle and was unable to remember the precise mechanism of injury. He visited his local hospital where he was diagnosed with a navicular fracture and treated with a short-leg cast for six weeks. He was referred to our institution because of nonresolution of his prolonged foot pain six months after the initial injury.

**Figure 1:**
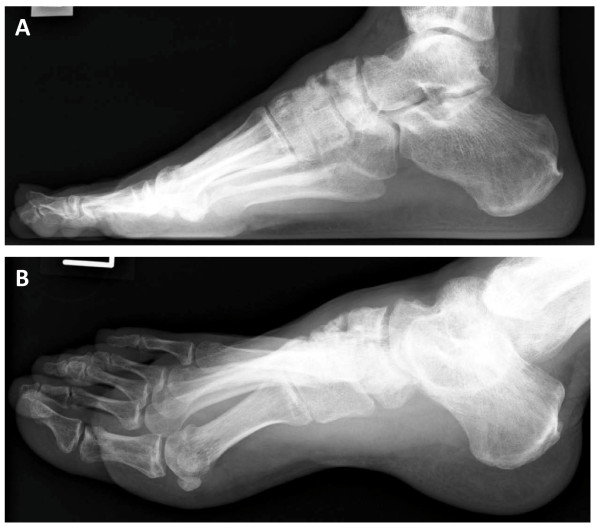
**Radiographs at the time of presentation showed atrophic bones and non-union of navicular fragments**. **(A) **Lateral radiograph. **(B) **Oblique radiograph.

**Figure 2:**
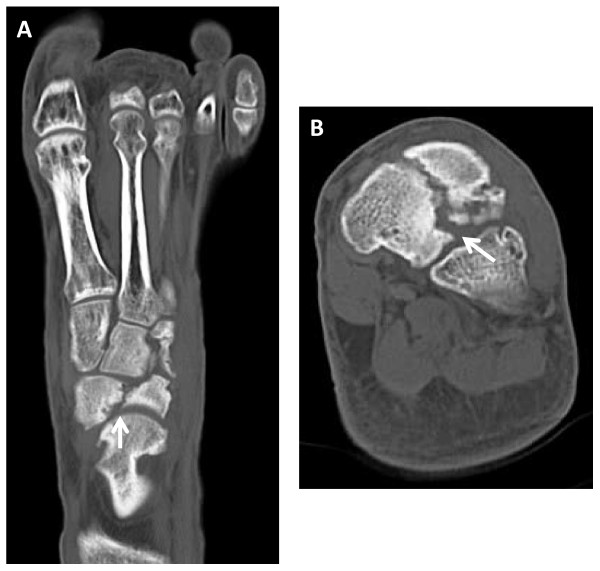
**Computed tomography scans at the time of presentation**. **(A) **Axial image. **(B) **Coronal image.

**Figure 3:**
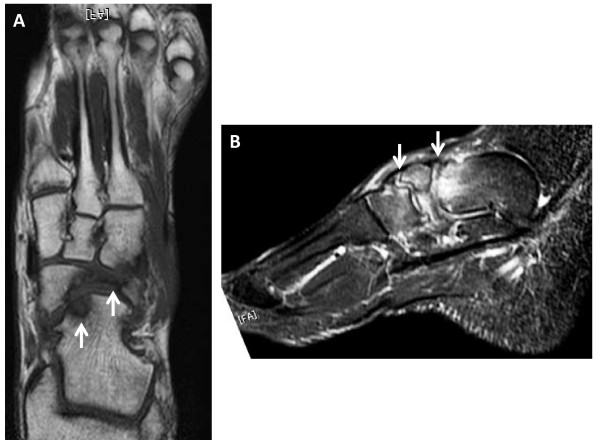
**Preoperative magnetic resonance images. (A)** Axial T1-weighted image showed avascular necrosis of the navicular bone and existence of a cystic lesion in the talar head. **(B) **Sagittal T2-weighted image showed slightly ragged talonavicular joint cartilage and incongruity of the talonavicular and navicular-cuneiforms joints.

Physical examination revealed that his left foot was slightly swollen, but the skin color was normal and there was no local heat on his instep. Blood tests did not indicate infection or inflammatory disease. There was tenderness over his middle foot, and he had active toe plantar flexion and dorsal flexion without restriction. Injections of lidocaine on the navicular bone resulted in temporary resolution of pain. Radiographs and CT scans revealed that navicular bone union had not been obtained and the fragments were atrophic. There was also associated incongruity of the cuneonavicular joint (Figure 1 and 2). T1-weighted MRI showed low-signal intensity on the lateral fragment, and T2-weighted MRI revealed high-signal intensity on the talonavicular joint and a slightly ragged joint cartilage, and a homogenous high-signal intense cystic lesion in the talus (Figure 3). We suspected there was avascular necrosis of the navicular bone fragment, and primitive osteoarthritis of the talonavicular joint because of the preserved joint space.

**Figure 4:**
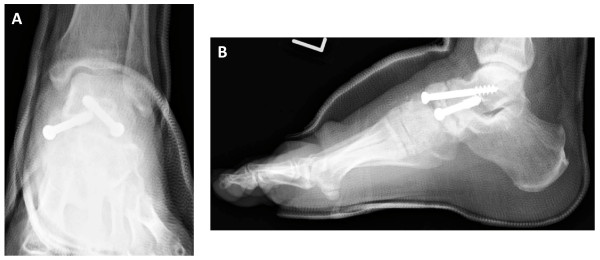
**Postoperative radiographs showed fixation with two screws**. **(A) **Anteroposterior radiograph. **(B) **Lateral radiograph.

The surgical technique used two longitudinal incisions on the lateral and medial fragment of the navicular bone. The gap of pseudarthrosis was filled with soft tissue and there was no movement between the two fragments. The talonavicular joint capsule was opened widely to expose the articular surfaces of the talar head and proximal navicular. The articular cartilage was damaged and partially eburnated. After opening the joint capsule we removed the remainder of the cartilage from the articular surface of the talar head and navicular bone by roughening the subchondral bone using a shaver and chisel. Compression and fixation of the talonavicular joint were achieved using two 6.5 mm partially threaded cancellous screws (SYNTHES), which were both inserted in a distal- to- proximal orientation (Figure 4). Postoperatively, a short leg cast was applied for six weeks. Progressive weight-bearing as tolerated was allowed after removing the cast.

**Figure 5:**
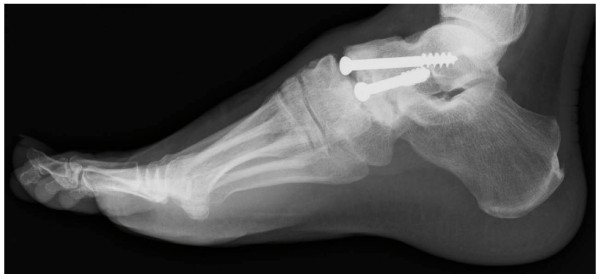
**Lateral radiograph at one year after the operation showed completely united talonavicular joint**.

The patient was followed clinically and radiographically at regular intervals, and one year after the operation, joint fusion was radiographically obtained, and he reported no pain or disability (Figure 5).

## Discussion

There are few cases in the literature describing the result of isolated talonavicular arthrodesis in patients with traumatic arthritis [3,6]. Most of the literature addresses patients with either inflammatory arthritis or adult acquired flat foot [4,5,7-11]. Main reported that the fracture of navicular was caused by shearing forces between the cuneiforms and the talar head, and triple arthrodesis was effective for persistent symptoms [12] while Chen maintained that isolated talonavicular arthrodesis provided both pain relief and functional improvement in traumatic arthritis [3].

In our case, images revealed aspects of osteoarthritis of the talonavicular joint and pseudarthrosis of the navicular bone, and we suspected lidocaine penetrated to the talonavicular joint and the gap of pseudarthrosis. Therefore, we thought the middle foot pain was caused by both osteoarthritis and pseudarthrosis and we planned to perform both a fixation of the navicular bone fragments and arthrodesis of the talonavicular joint. In fact, the gap of navicular pseudarthrosis was filled with soft fibrous tissues. However, no abnormal mobility between navicular bone fragments was identified. In the talonavicular joint, the articular surface was partially eburnated, and osteophyte and bone cyst were found. We concluded that the middle foot pain was derived from osteoarthritis of the talonavicular joint, and performed isolated arthrodesis for the talonavicular joint. However, if abnormal mobility had existed between the two fragments of the navicular bone, we would have performed not only arthrodesis of the talonavicular joint but also fixation of the navicular bone fragments or additional bone transplantation. As a result, three months after the operation, the patient felt no middle foot pain and was able to return to work as a bus driver. One year after the operation, radiography showed that the talonavicular joint was fully fused.

## Conclusion

Isolated talonavicular arthrodesis is one of the effective procedures for the treatment of traumatic talonavicular arthritis due to an ununited navicular bone without abnormal movement both in providing pain relief and functional improvement.

## Consent

Written informed consent was obtained from the patient for publication of this case report and any accompanying images. A copy of the written consent is available for review by the Editor-in-Chief of the journal.

## Competing interests

The authors declare that they have no competing interests.

## Authors' contributions

NK performed the surgical procedure, examined the case file, reviewed the literature and made major contributions to the writing of the manuscript. TN, YYT, and YNT participated in surgery and contributed in the conception and design of the manuscript. TF, SH and MK also contributed to writing the manuscript and its preparation. All authors read and approved the final manuscript.
